# Gleanings from Our Exchanges

**Published:** 1873-02-15

**Authors:** 


### Gleanings from Our Exchanges.

The Electric Bath.—Alexander Murray, M.D. (*N. Y. Med. Journal*, Oct., 1872), states, from his experience and personal use of the electric bath during the last five years, he finds it a pleasant, invigorating, and useful adjunct in the treatment of diseases. He speaks with confidence, and without speculation, as to the beneficial effects in all forms of rheumatism and allied diseases, hysteria, chorea, paralysis, amenorrhœa, diseases of the bladder, cholera-morbus, cholera-infantum, and wasting diseases of children, etc. The length of time to remain in the bath should range from ten to twenty-five minutes. For children, five to ten minutes will be sufficient (Children are so delighted with its pleasant, exhilarating sensations, that it is difficult to get them to leave the bath). The best time to take the bath is between breakfast and dinner, or a short time before retiring to bed. It may be taken at any time when a person may not feel in his usual health, or during the prevalence of an epidemic, or an unusual electrical state of the atmosphere, but never when the stomach is full of food. There need be no apprehension of catching cold after taking the bath, by going out directly in the cold air. In using the galvanic current, and to avoid giddiness and other unpleasant sensations, the positive pole should be placed at the feet, and the negative at the head. To apply electricity to a patient while in the bath, the quantity of water in the bath-tub should be reduced, so that the upper surface of the body would be uncovered of water; then let the physician cover the body with a large towel well wet with the water of the bath, and commence the application of electricity at the head downward, over any part or parts of the body desired, by means of a large sponge-covered electrode connected with the positive pole of the battery. The patient should at the same time keep one or both feet pressed firmly against the metal plate at the foot of the tub.—*Med. Record.*

Experimental Researches on Pericarditis. — S. H. Chapman, M.D., of New York, records his observations on "Pericarditis," with twelve wood-cuts, in the October number of the *Amercan Journal of Medical Sciences*, as furnishing additional proof of the truth of the opinions held by Stricker in his controversy with Cohnheim. The specimens collected by him upon this subject during these studies were shown by Professor Stricker, in the course of his argument, for examination by the general assembly at Leipzig. The results of the investigations upon the inflamed pericardium are as follows: 1st. All cell elements of a tissue during inflammation multiply. 2d. New formation takes place, first, of cells; second, of connective tissue (false membranes); and, third, most probably of nerves.—*Medical Record.*

Oxytoxic Action of Quinine.—R. H. Rutland, M.D., of Las Animas, Colorado (*Am. Jour. Med. Sciences*, Oct. 1872), has for eighteen years prescribed quinia in large doses, regardless of the pregnant condition of the patient, and has never seen any unfavorable result. Indeed, in treating a patient suffering from malarial fever, and threatened abortion, he esteems quinia the surest remedy for averting it; not because it possesses any special power to procure uterine quietude, but because it would break up the morbid excitement upon which the uterine activity most probably depended. That quinia has no special oxytoxic properties in cases in which it is indicated, he is as well satisfied as of any therapeutic fact.

Septicæmia.—M. Davaine, in his second paper on Septicæmia, read at the Academy of Medicine on the 8th inst., stated that he proposed examining two questions, viz.: Does septicæmia, experimentally produced, invade all animals indiscriminately, or does it exert its special effects on certain species? And what is the condition which imparts an extraordinary virulence to the blood of an animal inoculated with a putrified substance?

His researches, already recorded, have established the fact that the intensity of the affection so produced is not proportionate to the volume or mass of the animal, but that it is in relation with the nature of (to use M. Claude Bernard's expression) its *milieu interieur*—*i.e.*, it is a question of species. Thus, while the rabbit is so extraordinary susceptible as to be speedily killed with the trillionth or quintillionth of a drop of virulent blood, the guinea pig, a smaller animal of very similar habits, does not always fall a victim to strong doses, and is proof against the small ones which kill the rabbit. Experiments, since performed on the rat, mouse, and fowls, further show the different susceptibilities of different species.—*Times and Gazette, Oct.* 19, 1872.

Intermediate Amputation. — Joseph W. Thompson, M.D., Paducah, Ky., (*Richmond and Louisville Medical Journal*, September, 1872), in cases of compound fracture of the lower extremeties, does not believe, as a general thing, in amputations in the intermediate stage. When the time for primary amputation, which is established as decidedly the most favorable stage, has passed, he thinks surgeons had better trust the dangers that may arise in the intermediate condition, be they ever so grave, and await to take the chances of secondary amputation. He is satisfied that if accurate statistics on this point could be had, they would show a better success in taking the chances of the patient passing through the perils of the intermediate period, and then making a secondary operation. He publishes two interesting cases, in connection with his remarks.

Chloroform in Eclampsia. — Benjamin Rhett, M.D., of Abbeville, S. C. (*Richmond and Louisville Medical Journal*, September, 1872), gives affirmative evidence of the value of the prolonged use of chloroform for allaying the irritability of the brain in cases of eclampsia; and does not hesitate to commend it to the attention of the profession, not as a curative, but controlling agent in this frightful malady.—*Medical Record.*

Honorary Fellows of the Obstetrical Society of London.—At a late meeting of the Obstetrical Society of London, the following gentlemen of New York city were elected Honorary Fellows of the Society : Dr. Fordyce Barker and T. Gaillard Thomas.

Mental Vigor in Old Age.—It is said of Arnauld, the Jansenist, that he wished his friend Nicole to assist him in a new work. Nicole replied : "We are now old ; is it not time to rest?" "Rest!" exclaimed Arnauld, "have we not all eternity to rest in."

The Inventor of Spectacles.—On a tombstone in Florence is this inscription :—"Here lies Salvino Armato d' Armati, of Florence, the inventor of spectacles. May God pardon his sins. The year 1318."

Dr. Austin Flint has been elected President of the New York Academy of Medicine.

